# Supplementary material for: Electrophysiological characteristics of non-pulmonary vein triggers excluding origins from the superior vena cava and left atrial posterior wall: Lessons from the self-reference mapping technique
Source: PLoS One. 2022 Apr 6;17(4):e0263938. doi: 10.1371/journal.pone.0263938 (PMC8985937; doi:10.1371/journal.pone.0263938)
Supplement: Attachment — Submitted filename: Response to Reviewers.docx [file pone.0263938.s002.docx]

**Response to Editor/Reviewer**

We appreciate your great advice for making our manuscript more sophisticated. We provided our responses to the reviewer’s comments in a blue non-bold font as follows. We hope this revision process has remedied all of your concerns with the original manuscript (OM). The changes and corrections of the OM are shown in a highlighted text in the revised manuscript (RM). We hope our manuscript will be accepted for publication with this version.

Editor Comments

1. How did the authors ensure that PVs were not associated with the AF initiations? Did the authors place PV catheters during determination of the AF origin?

→Thank you for your comment. Before AF induction, we performed PV isolation. Moreover, if the earliest activation site was near the PV, we placed a catheter inside the PV isolation line and checked whether PV isolation remained.

2. How could the authors draw 3D-maps during the initiation of AFs? Did the author also use non-contact mapping to identify the origin (i.e., snap shots)? I think it is difficult to draw 3D-maps during initiation (i.e., only one beat).

→Thank you for your comment. I completely agree with your opinion. We used the self-reference mapping technique, reported in Reference #10. In this technique, we don’t draw an activation map. We place a tag at the earliest activation site among a multi-electrodes catheter, and using the earliest activation site as a reference, we move the multi-electrodes catheter to find earlier activation site as shown in the following figure. This figure was reported in Reference #10.

3. Did the authors check voltage maps around the origin of AF? In Fig 1, the signal amplitudes at PEN15-16 keep changing. How were the unipolar signals at the origin of AF?

→Thank you for your comment. We did not make voltage maps. However, as shown in “Limitations” section, the voltage assessment at the non-PV-SVC-LAPW trigger must be accurate because this mapping provided high density map around the origin and in fact non-PV-SVC-LAPW triggers were all eliminated by the RF application in a small area.

As you kindly pointed out, voltage maps were not able to obtain during AF storm, non-regular sinus rhythm, like Fig1. Therefore, we evaluated the sinus voltage just before AF initiation. The unipolar voltage was not recorded during the procedure.

According to your suggestion, we added the following sentences.

Page 8, Line 9-10

“The sinus voltage was assessed at the last sinus rhythm, just before AF initiation.”

Page 18, Line 9-7

“Fifth, the unipolar signals were not recorded during the procedure and not assessed in this study.”

4. If the authors believe these AFs were not associated with PVs, why did the authors isolate PVs?

→Thank you for your comment. According to the guideline (2017 HRS/EHRA/ECAS/APHRS/SOLAECE expert consensus statement on catheter and surgical ablation of atrial fibrillation), PV isolation is the most important first step to treat AF with ablation. Therefore, we performed PV isolation before AF induction in all cases.
